# Essay on Trembles or Animal Poison

**Published:** 1850-01

**Authors:** J. M. Logan

**Affiliations:** Palestine, Illinois


### ARTICLE II.

*Essay on Trembles, alias Animal Poison---A Paper read before the Æsculapean Society*---By Dr. J. M. Logan, of Palestine, Illinois.

I have selected this endemic of the West as the subject of the present essay, rather for the purpose of eliciting inquiry, observation and facts relative to the cause, nature and treatment of this disease, than of attempting to establish any preconceived opinion or theory of my own, or any that may have been previously published.

It is not deemed necessary to enter into a lengthened detail of arguments to prove the existence of this malady. A statement of a few facts is all that I will attempt here: and the first is, that there are certain locations in various parts of the western country, that have been from their first settlement, and still continue to be, subject to this peculiar disease; whilst other locations, hard by, have always been exempt. Second, wherever this disease prevails in the human species, the lower animals are liable to a peculiar and fatal disease called *Slows* or *Trembles*. Third, that in the human species the disease is of some specific poison, obtained, in most cases, from the milk or flesh of animals, which have fed on these infected lands. What is the local cause or specific virus which invades these districts, inducing the disease, we do not know ; as no two that have experimented, or written upon this subject, agree. Some have attributed it to the *Indian Hachy;* some to one of the species of *Rhus*, and some to another ; some to the various other products of the vegetable kingdom. Others contend that it is of mineral origin; and others that is an aerial poison. But as long as the subject remains a matter of doubt, it becomes no one to affirm positively, that the cause of this malady is or is not vegetable, 'mineral,' or aerial poison. It is certainly true that animals feeding on lands infected, at certain seasons, under certain circumstances, will be attacked with the disease ; but this does not prove that it is of vegetable origin : nor does the fact that this disease prevails most generally during, or immediately after a drought, prove that it is of mineral origin, or in the water.

But that this disease is not a species of congestive fever, generated by peculiar atmospheric miasma, may rather be inferred from the minute similarity of all the various cases, occurring at all periods of the year, under very varied circumstances ; and from the facts that in nearly all the cases in the human subject, the cause can be traced to its animal origin ; and that this disease is only known in the region of country where animals are subject to the *Slows.*

The strongest argument for the vegetable origin of the virus producing this disease, is the known fact that carniverous animals have the disease only after preying upon the carcasses of graminiverous animals that have died of the disease.

I have seen several cases caused by eating beef, and butter; and still more by drinking milk ; and I have witnessed only two cases where the poison had not been from using either; and of these cases I have never been satisfied as to the cause. They lived in an infected region, but had no cow ; and in answer to interrogations, stated they had not eaten beef or butter, as they were unable to buy : but it has been suggested to me that they might have eaten milk with the neighbors; for during the same season there were some twenty head of cattle died within a mile of them, and some three or four cases of the disease that could be clearly traced to animal origin.—In one instance, to illustrate this fact, Mr. W., whose wife had had the disease the previous fall, put up his cow in a small lot near his house, fed her on corn, and watered her from his well. After she had been up some weeks, Mr. W. was attacked with the disease very violently, and two days after, two of his children were taken down, and in two or three days after, two more were taken down. They were then advised to cease milking the cow, which appeared healthy. The cow, in a few days, began to show marks of disease, and died in a week. The dogs that preyed upon the carcass had the *slows*, and some died. Buzzards that fed upon it were unable to fly; thus proving that the cause of the disease was derived from the milk and flesh of the cow. In the meantime Mrs. W. was taken violently, and the other members of the family were all more or less affected.

Numerous other cases could be cited, proving the same point; but I will not impose upon the patience and time of the society so far as to narrate them; but will proceed to the diagnosis and treatment of this disease, which to the practitioner are more interesting and important.

There are two forms in which we meet with this disease; the acute and sub-acute or chronic. To the first the name of *milk-sickness* is usually given, and to the latter, the appropriate title of *slows*. They are, however, the same disease, produced by the same cause, and each form liable to be transformed into the other, and differing only in degree. In the sub-acute form, the individual is languid, unable to make any exertion of body or mind, appetite variable, bowels rather torpid, palpitation of the heart, some degree of stiffness of the limbs, trembling, and sickness of stomach if any considerable exertion is made, or if taking food is deferred beyond the usual time. This state of things may exist for months after the cause is once introduced, unless removed by the sanative efforts of nature, or by a proper course of remedial treatement: or it may be transformed into the acute form by long fatigue, fasting, or over exertion, and in some instances by neglecting the bowels, or suffering them to remain constipated for some days.

When the disease assumes the acute form, the individual is seized suddenly with nausea, faintness, and prostration: surface and extremities below the natural temperature—sometimes cold and clammy, great distress and anxiety depicted in the countenance, and the bowels almost universally constipated.

The heart and large arteries beat with violence, whilst the pulse at the wrist remains almost natural. Excessive vomiting, with insatiable thirst, and in most cases a craving for alcoholic liquors. No pain or tendernes over the region of the bowels. The tongue is in most cases but slightly coated, or changed from its natural appearance. Breathing is like that of a person under the influence of a nauseating dose of ipecac, or tartarized antimony. No vermicular or peristaltic motion of the bowels can be perceived. A complete retroverted action of the stomach comes on, and at every effort of vomiting a fluid is ejected of variable appearance; it is sometimes colorless, and at others it is like soap suds, and at others it is like indigo water, and in the last stage of cases that terminate fatally, it is of a dirty brown with a dark colored sediment. But in all it has the peculiar smell of the disease; and is generally represented by the patient as having a sweetish and most sickening taste.

Vomiting comes on at regular intervals, and is so violent that every article of diet, drink or fluid medicine is ejected. During the intermission of vomiting, the patient usually lies on his back, tossing his limbs about, and sometimes in a partially comatose state, but is at all times easily aroused when spoken to; and when interrogated as to his feelings, his only answer is, " O, I am so sick!"

The amount of fluid matter ejected in violent cases, is enormous; and in cases that terminate fatally, the irritability and retroverted action of the stomach continue until death closes the scene. In some cases the patient will occasionally complain of wandering pains in the limbs; and in some cases there is a cramping of the muscles of the limbs.

*Prognosis.*—When the disease is overcome by proper remedial agents, the heart and large arteries begin to return to their natural pulsations. The irritability of the stomach subsides, and the vermicular motions of the intestines can be perceived: the skin and extremities take on their natural heat, and after a thorough purgation of the bowels the patient generally recovers speedily.

The treatment of the chronic form of this disease, consists in purgatives and stimulants. In recent cases one or two doses of Cook's pills, followed with salts or oil. will be found sufficient, with stimulants; but in cases of long standing more purgatives will be required, and it will expedite the cure to begin with a few doses of calomel alone, or combined with rhubarb or jalap. In both forms of the disease alcoholic liquors can be taken in large quantities, without producing any intoxicating effects, and are of much importance as remedial agents. In the acute form of the disease, calomel in large doses, combined with opium, is most likely to be retained, and best allays the irritability of the stomach; and I have never been able to perceive that opium prevented the cathartic effect of calomel, but have thought that it rather promoted it. The calomel should be repeated every four or six hours until the bowels are evacuated; and in cases of extreme irritability, it will be best to give smaller doses and repeat them immediately after each attack of vomiting, so long as any of the medicine appears in the ejected fluid.

After the bowels have been moved, (and in cases of great prostration before,) spirits, brandy, whiskey, or hot toddy, should be freely administered. Sinapisms and blisters should be applied early in the disease, if we wish any benefit from them. I have bled in a few cases, but never with any marked advantage. Stimulating injections are often serviceable in promoting the action of the bowels.

When the stomach will bear it, a full dose of castor oil, combined with spirits turpentine will act beneficially after calomel. In one or two cases I have removed the constipation with 20 grs. Tartarized Antimony dissolved in a quart of warm water, as an injection, after calomel, salts, croton oil. and oil and turpentine had proved ineffectual.

After the bowels have been partially restored to their natural action, flowers of sulphur is preferable as an evacuant, to the neutral salts or oil. In some instances, when the constipation of the bowels is overcome, and the vomiting ceases, there is a strong tendency to a low typhoid state, requiring the free use of tonics and stimulants; and in some rare cases reaction supervenes, requiring an active course of treatment, which cases should be treated according to the indication of prevailing symptoms.

During convalescence the bowels should always be kept open by mild laxatives, and great care taken not to overload the stomach, as more relapses occur from the neglect of these than all other causes. I have seen no theory as to the *modus operandi* of this virus, and it may not be deemed out of place for me to speculate on this point, leaving the field open for your views, criticism and remarks. And first it does not act on the stomach like lead or arsenic, as its effect is not usually made manifest until it has been taken up by the absorbents; for in a large majority of cases the symptoms of disease are not developed for some days after the poison has been received into the stomach. The first symptoms of disease are not pain, or sickness at the stomach, or gastric derangement; but weakness, stiffness of the joints, and palpitaton of the heart. From this, we might infer that this virus, after being taken up into the circulation, acted specifically on the nerves of motion, producing a partial paralysis or weakening of the motor nerves and the capillary vessels, and stimulating the nerves of the heart and stomach, producing the violent action of the former, and the retroverted action of the latter. Thus it may be that cathartics cure, rather by stimulating the nerves of the intestinal canal, and exciting the natural secretions, than by removing the poison, which was the original cause of the disease.

Thence it is that the most powerful stimulants appear to act beneficially, notwithstanding the violent action of the heart and large arteries contra-indicate all stimulating remedies. It is our misfortune that popular prejudice is so averse to post mortem examinations; and consequently we are unable to speak from actual observation of the organs or tissues that are in a normal or diseased condition in this disease, and we must patiently await the time when public opinion will awake to the true interests of medical science and humanity.

